# Wicked Bodies: A Health and Well-Being Toolkit Addressing Eating Disorders Within LGBTQIA2S+ Communities

**DOI:** 10.1177/15248399221133734

**Published:** 2022-11-22

**Authors:** Phillip Joy, Olivier Ferlatte, Megan Aston, Hannah Minzloff, John Hillis

**Affiliations:** 1 Mount Saint Vincent University, Halifax, Nova Scotia, Canada; 2 École de santé publique, Univeristy of Montreal, Montreal, Quebec, Canada; 3 Dalhousie University, Halifax, Nova Scotia, Canada; 4 Truefaux Films, Dartmouth, Nova Scotia, Canada

**Keywords:** LGBT, eating disorders, film, arts-based methods, queer, teaching

## Abstract

Wicked Bodies is a toolkit for addressing eating disorder in LGBTQIA2S+ (lesbian, gay, bisexual, transgender, queer, intersex, asexual, Two-Spirit, and other sexual and gender minority) communities, an increasing prevalent issue that can have serious consequences on the health and well-being for LGBTQIA2S+ people. The toolkit consists of a series of short films and a discussion guidebook that provide a template that can be used for engaging with LGBTQIA2S+ youth through a lens of compassion and cultural humility. Wicked Bodies does this by presenting the lived experiences of a diverse range of LGBTQIA2S+ individuals navigating sociocultural pressures, gender expectations, and peer-based ideals around body weight and shape. Feedback from three screening events revealed that Wicked Bodies has the potential to be transformative as a health promotion initiative.

## Assessment of Need

Eating disorders are characterized by severely disturbed eating behaviors and, within youth, have been reported to be associated with the development of anxiety disorders, depression, substance use, and self-harm behaviors (McClain & Peebles, 2016). Lesbian, gay, bisexual, transgender, queer, intersex, asexual, Two-Spirit, and other sexual and gender minority (LGBTQIA2S+) adolescents are at an increased risk of eating disorders and disordered eating behaviors (Parker & Harriger, 2020). Approximately 54% of LGBTQIA2S+ adolescents have been diagnosed with a full syndrome eating disorder during their lifetime, with an additional 21% suspecting that they had an eating disorder at some point during their life (Parker & Harriger, 2020). The causes are still not fully understood, but researchers suggest that societal and cultural standards for bodies and hegemonic ideals of femininity and masculinity can influence eating disorders and disordered eating practices (Nagata et al., 2020), as well as minority stress (Parker & Harriger, 2020). McClain and Peebles (2016) note that most studies on disordered eating have been centered on cisgender individuals. This often creates challenges in accessing and utilizing health services for LGBTQIA2S+ people and contributes to health disparities (Smalley et al., 2016). As Parker and Harriger (2020) further emphasize, it is “vital to enhance community resilience (equipping the community to provide resources and support for sexual and gender minority individuals such as hotlines, support groups, role models, and policies and laws that advocate for LGBT individuals)” (p. 15).

## Description of the Innovation

Service providers like Eating Disorders Nova Scotia, Body Brave, and the National Eating Disorder Information Centre (personal communication, May 2022) note that most services available are created with cis-heteronormative considerations that are aimed at straight White women. These organizations have noted an important gap in the resources available for them to use for their LGBTQIA2S+ clients (personal communication, May 2022). To address this gap, our team designed and created a pilot community-informed resource called *Wicked Bodies—*a free digital resource toolkit that includes a series of short films and a discussion guidebook. Two film episodes were created. The first was an introduction to eating disorders within LGBTQIA2S+ communities. Table 1 provides an overview of the questions asked to the participants and the content discussed in Film 1: An Introduction to *Wicked Bodies*.

**Table 1:** Sections Included in Film 1

Introduction of participants	This includes their pronouns and identities
What does your eating disorder look like?	This section discussions some of the symptoms of participants’ eating disorders
When did your eating disorder start?	The participants provide insight into when their eating disorders began
Sexuality and eating disorder	This section examines the connections between sexuality and eating disorders for the participants
Media Influences	Participants discuss how media and social media have influenced their experiences.
Identity and treatment	This section explores how participants’ identities shaped their treatment.
Acceptance, care, and improved supports	Participants discuss finding competent doctors, importance of acceptance and understanding, and their thoughts on how to improve care practices.
The journey of recovery	Discussions of the participants’ recovery

The second episode focused on similar questions and topics but specifically in the context of trans and nonbinary folks living with eating disorders.

## Intended Impact

*Wicked Bodies* is designed to help reduce stigma, generate hope, and invite more open and nuanced conversations about disordered eating, eating disorders, and body dysmorphia within LGBTQIA2S+ communities through uplifting narratives. Wicked Bodies is intended to be used by treatment and health care centers, schools and higher education institutions, nonprofit programs working with LGBTQIA2S+ clients, and libraries and community groups.

## Evaluation Approach

We had three film screening and discussion events to evaluate our first two completed episodes. One event was virtual and two were in-person (Montreal and Halifax, Canada). We distributed and collected feedback surveys at these events. Of the attendees, 30% completed the survey, of which 44% were university students, 31% were health professionals, 14% were people living with an eating disorder, 8% were educators, and 3% were community service providers.

## Challenges and Successes

The main challenge in creating *Wicked Bodies* was obtaining a diverse representation of various LGBTQIA2S+ groups. This was addressed by recruiting nationally. Our feedback indicated that the *Wicked Bodies* toolkit resonated with attendees noting that they appreciated hearing the varied perspectives of others, felt we created a safe space to view and discuss the films, felt a sense of connection with the people in the film, and that they could identify with the stories in the episodes. Attendees also appreciated the opportunity to share their thoughts, some of which are given in the quotes below:

I was also so happy to see the discussion about the difficulties accessing appropriate health care as this is so often misunderstood by members outside of the community. This gap/lack of services is particularly difficult in specialty areas like Eating Disorder care. (Quote from Feedback Survey)

The fact that the interviewees were positioned/presented as the experts on their experiences. The intimate nature of the film and the focus on people’s experiences. (Quote from Feedback Survey)

The characters are engaging, the themes relevant. It’s a very dark, deep theme, which is approached in a nondramatic or sensational way. (Quote from Feedback Survey)

## Next Steps and Lessons Learned

Our feedback also indicated that the individual stories of the people in the films need to be expanded so that viewers can more readily connect with the film participants. More episodes in this series would help to add depth to the film participants’ stories.

## Implications for Practice

The power of film and personal stories within health promotion efforts for LGBTQIA2S+ youth is highlighted through this project. It is important, however, to recognize that not all 2SLGBTQIA+ youth are affected equally, nor have the same concerns relating to their bodies and eating disorders (Nagata et al., 2020; Parker & Harriger, 2020). Our feedback supports this observation as many of the feedback surveys indicated that diversity within the project was critical to capture the nuances of the different communities within the rainbow acronym. Other considerations noted by our survey for health promotion initiatives relevant for LGBTQIA2S+ youth include implementing a community-informed approach and the use of first-person style storytelling. Film and discussion-based approaches to health promotion can be an engaging means to tell the lived experiences and first-person narratives of 2SLGBTQIA+ youth. Such methods can also be a way to create discussions about the complex nature of the world and its realities (Hearing & Jones, 2018), as well as ensuring youth feel validated in their identities. This is exemplified in the following quote from our survey:

Thank you SO much for doing this! As the only queer healthcare working on my team I often feel pressure to be the constant advocate / keeper of all knowledge and resources re: LGBTQIA2S+ experiences. Having access to these amazing resources will be supportive for our team to learn more and better support our clients/participants. I think many of the participants we work with will find the videos and other resources incredibly validating and help them know they aren’t alone in their experiences. This work is so important! (Quote from Feedback Survey)

We hope to provide access to *Wicked Bodies*, including our first film and the discussion guidebook freely through our website (https://phillipjoy.ca/wicked-bodies.html) to anyone we can use the resource, and to obtain funding for future episodes.
